# Impact of monotherapy on HIV-1 reservoir, immune activation, and co-infection with Epstein-Barr virus

**DOI:** 10.1371/journal.pone.0185128

**Published:** 2017-09-19

**Authors:** Maria Raffaella Petrara, Anna Maria Cattelan, Lolita Sasset, Riccardo Freguja, Francesco Carmona, Silvia Sanavia, Marisa Zanchetta, Paola Del Bianco, Anita De Rossi

**Affiliations:** 1 Department of Surgery, Oncology and Gastroenterology, Section of Oncology and Immunology, AIDS Reference Centre, University of Padova, Padova, Italy; 2 Division of Infectious and Tropical Diseases, Azienda Ospedaliera and University of Padova, Padova, Italy; 3 Division of Infectious Disease, Azienda Ospedaliera of Rovigo, Rovigo, Italy; 4 Istituto Oncologico Veneto (IOV)-IRCCS, Padova, Italy; University of North Carolina at Chapel Hill, UNITED STATES

## Abstract

**Objectives:**

Although monotherapy (mART) effectiveness in maintaining viral suppression and CD4 cell count has been extensively examined in HIV-1-infected patients, its impact on HIV-1 reservoir, immune activation, microbial translocation and co-infection with Epstein-Barr Virus (EBV) is unclear.

**Methods:**

This retrospective study involved 32 patients who switched to mART; patients were studied at baseline, 48 and 96 weeks after mART initiation. Thirty-two patients who continued combined antiretroviral therapy (cART) over the same period of time were included in the study. Markers of HIV-1 reservoir (HIV-1 DNA and intracellular HIV-1 RNA) were quantified by real-time PCR. Markers of T-(CD3^+^CD8^+^CD38^+^) and B-(CD19^+^CD80/86^+^ and CD19^+^CD10^-^CD21^low^CD27^+^) cell activation were evaluated by flow cytometry. Plasma levels of microbial translocation markers were quantified by real-time PCR (16S ribosomal DNA and mitochondrial [mt]DNA) or by ELISA (LPS and sCD14). EBV was typed and quantified by multiplex real-time PCR.

**Results:**

At baseline, no differences were found between mART and cART groups. Three (10%) mART-treated patients had a virological failure *vs* none in the cART group. Levels of HIV-1 DNA, intracellular HIV-1 RNA and EBV-DNA remained stable in the mART group, while decreased significantly in the cART group. Percentages of T- and B-activated cells significantly increased in the mART-treated patients, while remained at low levels in the cART-treated ones (p = 0.014 and p<0.001, respectively). Notably, levels of mtDNA remained stable in the cART group, but significantly rose in the mART one (p<0.001).

**Conclusions:**

Long-term mART is associated with higher levels of T- and B-cell activation and, conversely to cART, does not reduce the size of HIV-1 reservoir and EBV co-infection.

## Introduction

The standard recommended regimens for the treatment of HIV-1 infection consists in a combination of three antiretroviral agents; a boosted protease inhibitor (PI) or non-nucleoside reverse transcriptase inhibitor (NNTRI) is, in fact, commonly prescribed in combination with two reverse transcriptase inhibitors (NRTI). This combined antiretroviral therapy (cART) has dramatically changed the course of HIV-1 infection in a chronic disease, requiring lifetime HIV-1 therapy. The prolonged use of cART is nevertheless associated with side effects, including metabolic effects, lipodystrophy, drug-drug interactions, and long-term adherence problems.

Aiming to reduce the risk of long-term toxicity, to enhance medication adherence, to improve quality of life, and to contain treatment costs, several studies have been and are being carried out to examine the effectiveness of simplified strategies based on a protease inhibitor monotherapy (mART). The effect of mART on plasma viremia and CD4 cell count has been extensively studied [1–7]. Several studies have reported a higher proportion of mART-treated patients who have experienced viral rebound; however, the plasmaviremia was rapidly and easily suppressed by re-introduction of cART and without apparent long-term consequences [2, 4–7]. Conflicting results have nevertheless been reported regarding effectiveness of mART in controlling HIV-1 reservoirs or in penetrating “sanctuaries” such as the central nervous system, linked to a high risk of neurocognitive impairment [8,9]

The reduced control of HIV-1 replication in mART-treated patients may be due to smaller reduction in the size of viral reservoirs and/or increased activation/inflammation status, a hallmark of HIV-1 infection [10]. However, only a few studies have investigated these aspects in mART-treated patients. Two studies reported no significant differences in HIV-1 DNA levels between mART and cART, despite a higher proportion of intermittent viremia in the first group of patients [11,12]. An other study suggested that monotherapy *per se* does not impact HIV-1 DNA load [13].

Little is known about the immune activation of T cells. No difference was found in HLA-DR T-cell expression between mART- and cART-treated patients [14], while an increased cellular expression of HLA-DR^+^CD38^+^ on CD8^+^ T-cell has been reported in patients with virological failure [15]. Although immune activation may also result in chronic B-cell stimulation and expansion of EBV-infected cells [16], no studies have simultaneously investigated size of viral reservoirs, T- and B-cell activation and dynamics of EBV infection in mART-treated patients.

The aim of the present study was to evaluate the impact of monotherapy on HIV-1 reservoir, immune activation, microbial translocation and EBV co-infection in HIV-1 infected patients who were switched from cART to mART.

## Materials and methods

### Patient characteristics

In this retrospective study, a total of 64 (43 male and 21 female) HIV-1-infected patients attending the Infectious Diseases Division of Rovigo Hospital were studied. Thirty-two patients >18 year old with documented history of HIV-1 infection, stable cART, undetectable plasmaviremia (HIV-1 RNA <50 copies/ml) and good immunological status (CD4 >350 cell/μl and nadir CD4 >100 cell/μl) for at least 6 months were switched from cART to mART with one PI (Lopinavir/ritonavir). The main exclusion criteria from mART were previous virological failure during PI-based treatment, pregnancy, no-adherence to outpatient appointments, positivity to Hepatitis B antigens, and presence of mutations conferring resistance to PI (i.e 32I, 33F, 46 I/L, 47 A/V, 50V, 54A/L/M/S/T/V, 76V, 82A/F/S/T, 84V, 90M). All the patients underwent standard clinical and laboratory monitoring. Samples collected at baseline (i.e., within one week pre-switch from cART to mART) and at 48 and 96 weeks after mART initiation were studied. A control group of 32 patients, who had been receiving an unmodified PI-based cART regimen over the same period of time, was included in the study. Samples from these patients who continued cART were collected at baseline (i.e., at entry into the study) and at 48 and 96 weeks follow-up. The study was performed in accordance with the Helsinki Declaration and was approved by the Ethics Committee of the Istituto Oncologico Veneto, Prot. 12855, 2011/57.

### Sample collection

Peripheral blood mononuclear cells (PBMC) were obtained using Ficoll Hypaque gradient separation. PBMC and plasma samples were cryopreserved and respectively stored in liquid nitrogen and at -80°C until they were used.

### Quantification of HIV-1 reservoir

Levels of HIV-1 DNA were quantified in PBMC by real-time PCR, as previously described [17]. The results were expressed as HIV-1 DNA copies/10^6^ PBMC.

To quantify intracellular HIV-1 RNA, RNA was extracted from 3x10^6^ PBMC using Trizol Reagent (Invitrogen, Carlsbad, CA, USA). Five hundred μl of Trizol and 7 μl of QS, an internal control (Roche Diagnostic Systems, Branchburg, NJ), were added to the PBMC. The samples were incubated with 200 μl of chloroform for 15 min on ice. After centrifugation, RNA was recovered and stored at -20°C overnight with cold isopropanol. The samples were then centrifuged and the supernatant was removed. Each RNA pellet was then resuspended with 75 μl of elution buffer heated at 70°C. HIV-1 RNA levels were determined using the Amplicor HIV-1 Monitor Test (Roche Diagnostic Systems, Branchburg, NJ) and the Cobas TaqMan48 (Roche Diagnostic Systems, Branchburg, NJ) [18,19]. After the dilution factor was applied, the results were expressed as HIV-1 RNA copies/10^6^ cells.

### Flow cytometry analysis

Approximately 250,000 PBMC were stained with monoclonal antibodies (Becton-Dickinson, San Diego, CA, USA): anti-CD3 [fluorescein isothiocyanate (FITC)], anti-CD8 [peridinin chlorophyll protein (PerCP)] and anti-CD38 [phycoerythrin (PE)]; and anti-CD19 [peridinin chlorophyll protein (PerCP)], anti-CD10 [phycoerythrin (PE)-TexasRed], anti-CD21 [fluorescein isothiocyanate (FITC)], anti-CD27 [phycoerythrin (PE)-Cy7], anti-CD80 [allophycocyanin-H7 (APC-H7)] and anti-CD86 [allophycocyanin (APC)]. Appropriate isotype controls (mouse IgG1-PE and mouse IgG2b-APC) were used to evaluate non-specific staining. All the samples were analyzed by LSR II cytofluorimeter (Becton-Dickinson). A total of 50,000 events was collected in the lymphocyte gate using morphological parameters (Forward and Side scatter). Data were processed with FACSDiva™ Software (Becton-Dickinson) and analysed using Kaluza^®^ Analyzing Software v.1.2 (Beckman Coulter, Fullerton, CA, USA).

### Quantification of microbial translocation markers

A quantitative method based on real-time PCR assay was performed to quantify 16S ribosomal DNA (rDNA) with the primer pair and probe as previously described [18]. Results were expressed as 16S rDNA copies/μl plasma. Plasma levels of mitochondrial DNA (mtDNA) were quantified by real-time PCR using primer pair and probe as already described [20]; results were expressed as mtDNA copies/μl plasma. Levels of human soluble CD14 (sCD14) were determined by Quantikine Human sCD14 Immunoassay (R&D Systems Inc. Minneapolis, MN, USA), and results were expressed as sCD14 pg/ml plasma. Lipopolysaccharide (LPS) was determined in plasma samples diluted five-fold with endotoxin-free water and then heated to 70°C for 10 min to inactivate plasma proteins; LPS was then quantified using a chromogenic assay (Limolus Amebocyte Lysate QCL-1000), as previously reported [21], and results were expressed as LPS pg/ml plasma.

### EBV-DNA typing and quantification

A quantitative method, based on multiplex real-time PCR assay, was employed to quantify EBV type 1 and EBV type 2 in PBMC, as described elsewhere [22]. The results were expressed as EBV-DNA copies/10^6^ cells.

### Statistical analyses

Samples in which HIV-1 DNA and EBV-DNA were under the detection value (10 copies/10^6^ cells), were assigned the value of 10 to include them in the statistical analysis. The non-parametric Wilcoxon's rank sum test was used to verify the difference between cART and mART at baseline and during follow-up, whereas the paired comparisons within each therapy group between baseline measures and repeated measurements during follow-up were performed using the Wilcoxon's signed rank test. The level of statistical significance was set at p<0.05 and no correction for multiple comparisons were applied. All statistical tests were two-sided. Data were analyzed using SAS version 9.2.

## Results

### Characteristics of study population

Patient characteristics are listed in Table 1. At baseline the demographic and clinical characteristics of the two patients groups were similar. The majority of the patients had a long history of HIV-1 infection and cART treatment; 22(69%) cART and 24(75%) mART patients were taking emtricitabine/tenofovir, and 10(31%) cART and 8(25%) mART patients were taking abacavir/lamividune. Patients were with undetectable HIV-RNA for a median time of 41 (cART group) and 48 months (mART group). The patients were switched from cART to mART because of adverse events (41%) or to simplify treatment approach (59%).

**Table 1:** Characteristics of patients at baseline.

	mART (n = 32)	cART (n = 32)
Age, years*	45 (39–50)	45 (37–52)
Gender, n (%)		
Male	18 (56)	25 (78)
Female	14 (44)	7 (22)
Ethnicity/race, n (%)		
Caucasian	28 (88)	31 (97)
African	2 (6)	1 (3)
Asian	2 (6)	0
Risk behavior for HIV-1 infection, n (%)		
MSM^a^	10 (31)	18 (56)
ET^b^	19 (59)	11 (34)
TD^c^	3 (14)	3 (14)
Duration of HIV-1 infection-year*	7 (5–15)	6 (4–15)
CDC stage, n (%)		
A	23 (72)	20 (63)
B	8 (25)	11 (34)
C	1 (3)	1 (3)
Duration of cART-year*	6 (5–13)	5 (3–13)
Tenofovir-based cART, n (%)	24 (75)	23 (72)
Zenit HIV-1 RNA (log_10_ copies/ml)*	4.95 (4.55–5.50)	4.88 (4.51–5.04)
HIV-1 RNA undetectable, n (%)	32 (100)	32 (100)
Virological suppression (months)	48 (36–54)	41 (24–60)
Nadir CD4+ (cells/μl)*	298 (215–410)	327 (267–450)
%CD4*	18 (14–23)	16 (13–23)
CD4 count (cells/μl)*	690 (625–952)	634 (455–1051)

*Data are expressed as median (interquartile range- IQR).

^a^MSM: men who have sex with men.

^b^ET: heterosexual.

^c^TD: drug addicted.

During follow-up, no cART patients had virological failure, while one mART-treated patient had one HIV-1 RNA blip (defined as a transitory episode of HIV-1 RNA >50 copies/ml preceded and followed by plasmaviremia <50 copies/ml), one mART-treated patient had two HIV-1 RNA blips and 3 mART-treated patients had virological failure (defined as two consecutive HIV-1 RNA >50 copies/ml), at week 12 (1 patient) and at week 36 (2 patients) of follow-up. The patients regained virological control by adding the previous NRTI-backbone and were excluded from the mART group when they were returned to cART. No significant adverse effects and no deaths were observed in the two groups of patients during the study period.

### HIV-1 reservoir

**Fig 1:**
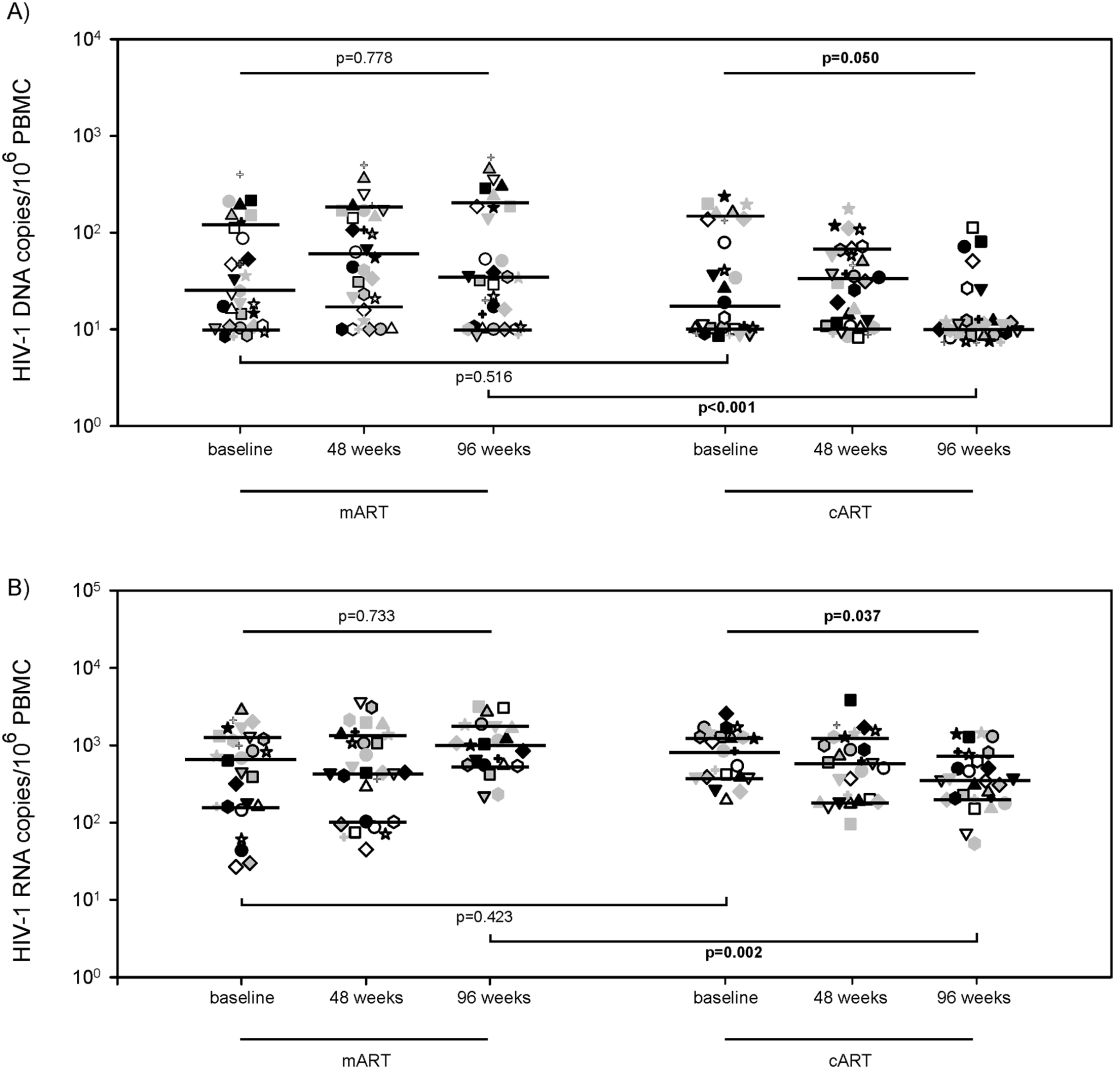
HIV-1 reservoir levels in mART-treated and cART-treated patients. HIV-1 DNA (A) and intracellular HIV-1 RNA (B) levels in mART and cART patients at baseline, at 48 and 96 weeks of follow-up. Each symbol represents one patient. The lines indicate the median and the 25–75th percentiles.

At baseline, the median (interquartile range-[IQR]) levels of HIV-1 DNA and intracellular HIV-1 RNA were similar in the two groups (Fig 1A and 1B). Overall, beyond individual patients’ variability, HIV-1 DNA levels remained stable in the mART group throughout the study period (24 [10–112], 55 [16–169], and 33 [10–186] copies/10^6^ PBMC at baseline (T0), week 48 (T48), and week 96 (T96), respectively; p = 0.778), but slightly decreased in the cART group (16 [10–137], 31 [10–59], and 10 [10–10] copies/10^6^ PBMC at T0, T48, and T96, respectively; p = 0.050) (Fig 1A). The intracellular HIV-1 RNA levels did not significantly vary in the mART group (680 [161–1318], 441 [102–1374], and 1035 [552–1836] copies/10^6^ PBMC at T0, T48, and T96, respectively; p = 0.733), while they decreased in the cART group (835 [385–1274], 595 [184–1261], and 358 [204–753] copies/10^6^ PBMC at T0, T48, and T96, respectively; p = 0.037) (Fig 1B). At the end of follow-up, the mART-treated patients had significantly higher levels of HIV-1 DNA and intracellular HIV-1 RNA with respect to the cART–treated patients (Fig 1A and 1B).

### Immune activation

**Fig 2:**
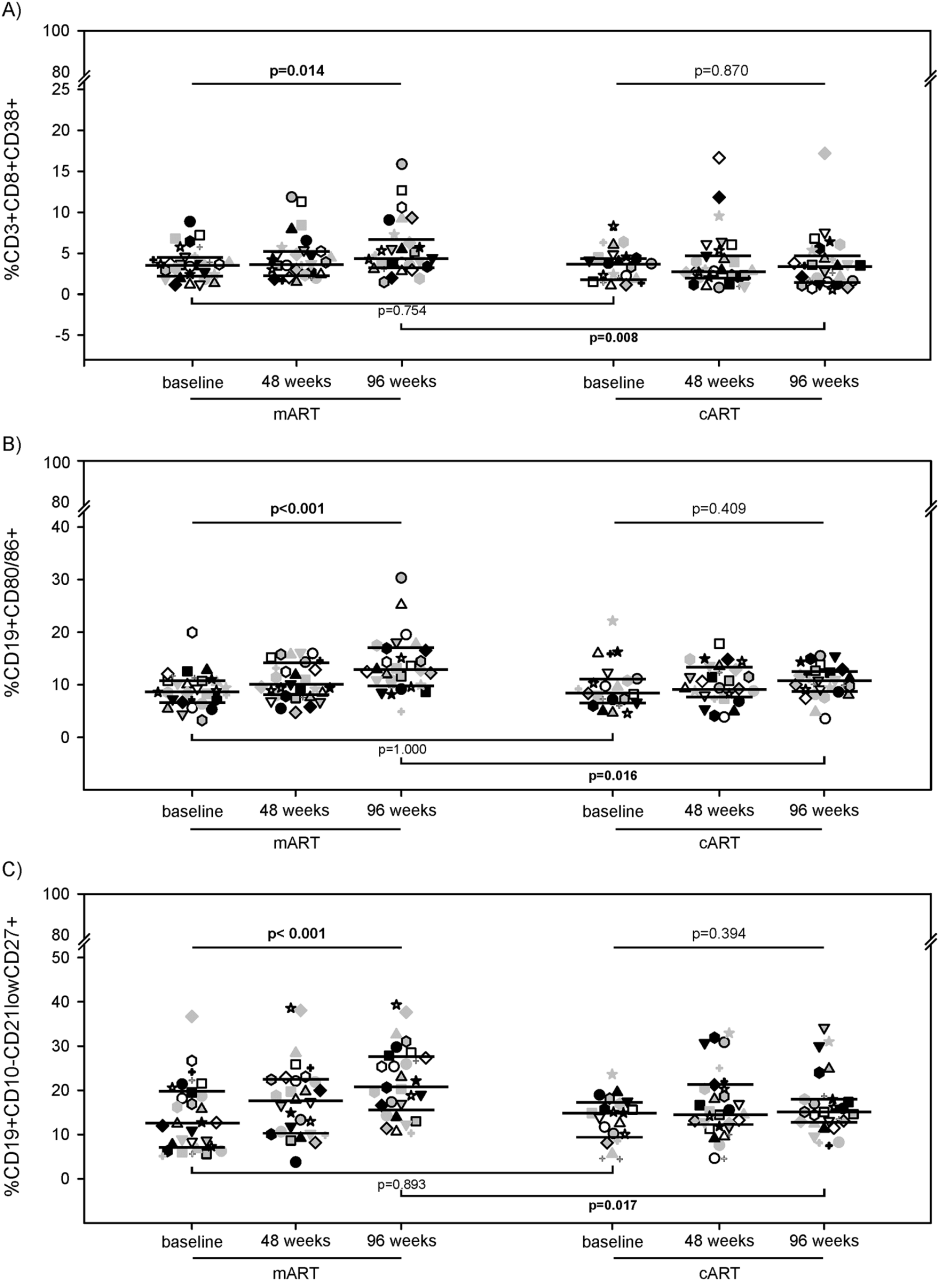
T- and B-cell immune activation levels in mART-treated and cART-treated patients. Percentages of activated T cells (A), B cells (B), and memory B cells (C) in mART and cART patients at baseline, at 48 and 96 weeks of follow-up. Each symbol represents one patient. The lines indicate the median and the 25–75th percentiles.

At baseline, percentages of activated T cells (CD3^+^CD8^+^CD38^+^) [21,23] were similar in the two groups (Fig 2A). The activated T cells significantly increased in the mART group during the follow-up (3.58 [2.31–4.60], 3.73 [2.36–5.33], and 4.46 [3.23–6.79] %CD3^+^CD8^+^CD38^+^ at T0, T48, and T96, respectively; p = 0.014), but they did not vary significantly in the cART-treated patients (3.72 [1.80–4.38], 2.81 [2.03–4.74], and 3.41 [1.54–4.85] %CD3^+^CD8^+^CD38^+^ at T0, T48, and T96, respectively; p = 0.870) (Fig 2A). At the end of follow-up, percentages of activated T cells were significantly higher in the mART-treated than in cART-treated patients (Fig 2A).

At baseline, activated B cells (CD19^+^CD80/86^+^) and activated memory B cells (CD19^+^CD10^-^CD21^low^CD27^+^) [24] were similar in the two groups (Fig 2B and 2C). During follow-up, the percentage of activated B cells increased in the mART-treated patients (8.63 [6.67–10.75], 9.96 [8.08–14.06], and 12.79 [9.76–16.74] %CD19^+^80/86^+^ at T0, T48, and T96, respectively; p<0.001), while they remained fairly stable within cART patients (8.38 [6.61–11.11], 9.38 [7.61–13.19], and 10.68 [8.79–12.59] %CD19^+^80/86^+^ at T0, T48, and T96 respectively; p = 0.409) (Fig 2B). A similar trend was observed for activated memory B cells. Indeed, percentages of these cells significantly increased in the mART group (12.56 [7.15–19.78], 17.64 [10.52–22.53], and 20.65 [15.42–27.59] %CD19^+^CD10^-^CD21^low^CD27^+^ at T0, T48, and T96, respectively; p<0.001), while they remained fairly constant in the cART group (14.82 [9.33–17.24], 14.46 [11.78–21.24], and 15.09 [12.40–17.94] %CD19^+^CD10^-^CD21^low^CD27^+^ at T0, T48, and T96, respectively; p = 0.394) (Fig 2C). At the end of follow-up, the percentages of activated and activated memory B cells tended to be higher in mART-treated than in cART-treated patients (Fig 2B and 2C).

### Microbial translocation

**Fig 3:**
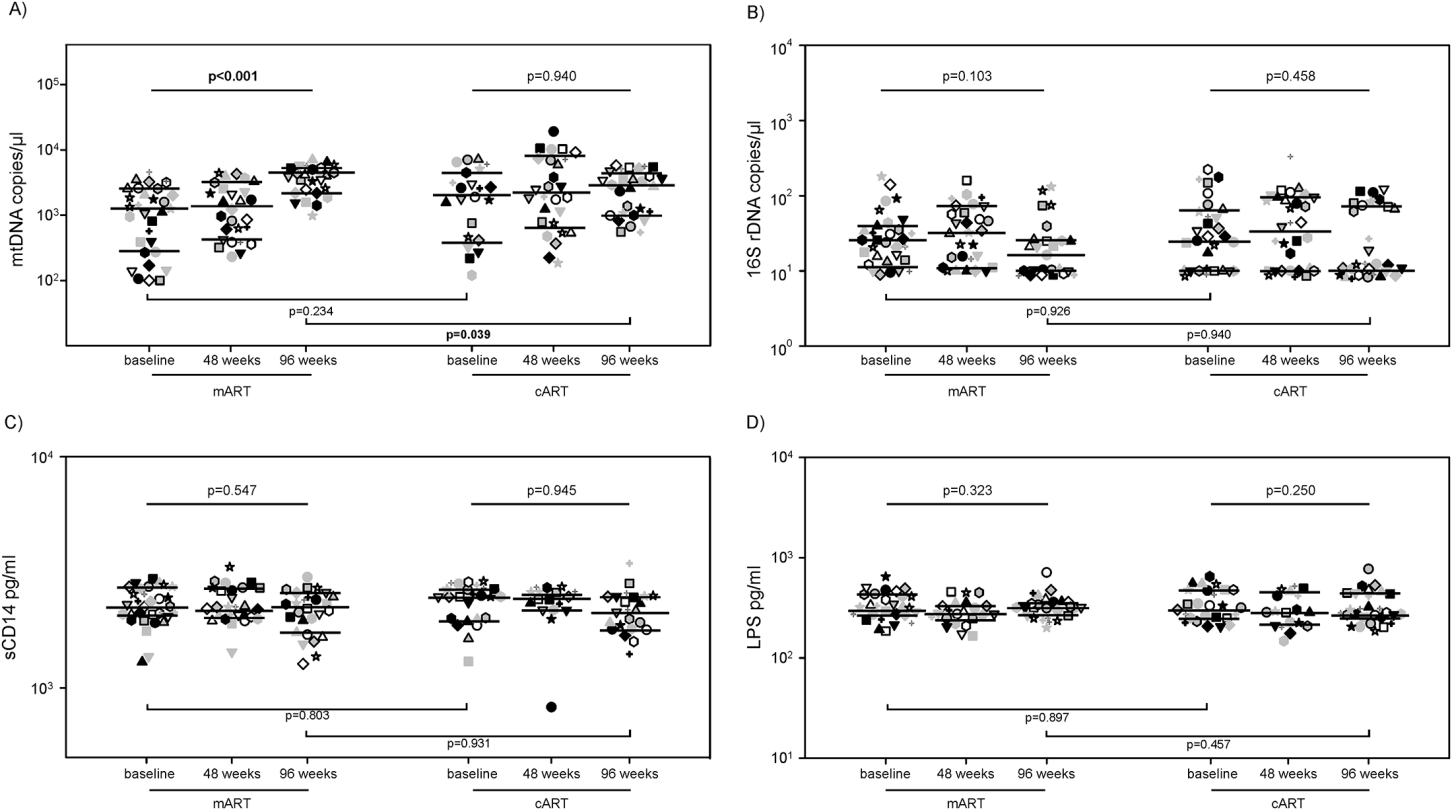
Circulating markers of immune activation levels in mART-treated and cART-treated patients. mtDNA (A), 16S rDNA (B), sCD14 (C) and LPS (D) levels in mART and cART patients at baseline, at 48 and 96 weeks of follow-up. Each symbol represents one patient. The lines indicate the median and the 25–75th percentiles.

At baseline, there were no differences in any of the microbial translocation markers studied (mtDNA, 16S rDNA, sCD14 and LPS) (Fig 3A, 3B, 3C and 3D). During follow-up, the levels of mtDNA significantly increase in the mART-treated patients (1254 [271–2546], 1221 [425–3153], and 4482 [2170–5213] copies/μl at T0, T48, and T96, respectively; p<0.001), while they did not significantly vary in the cART-treated patients (2030 [377–4424], 2213 [637–8104], and 2858 [983–4352] copies/μl at T0, T48, and T96, respectively; p = 0.940) (Fig 3A). No significant changes were observed in the other markers during the follow-up (Fig 3B, 3C and 3D). At the end of follow-up, mART-treated patients had significantly higher levels of mtDNA than cART-treated patients (Fig 3A).

### Co-infection with EBV-DNA

**Fig 4:**
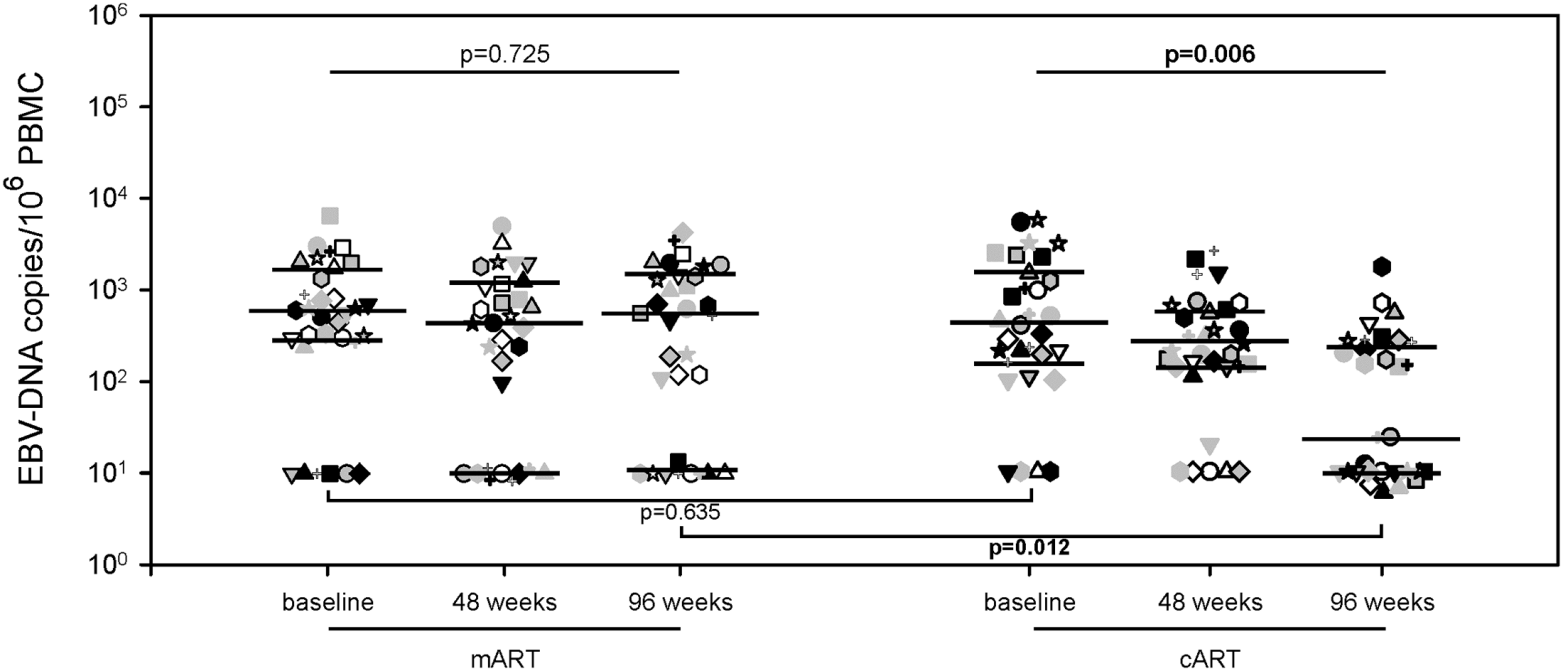
EBV-DNA levels in mART-treated and cART-treated patients. Levels of EBV-DNA in mART and cART patients at baseline, at 48 and 96 weeks of follow-up. Each symbol represents one patient. The lines indicate the median and the 25–75th percentiles.

At baseline, the levels of intracellular EBV-DNA were similar in the mART and cART groups (Fig 4). During follow-up, the levels of EBV-DNA did not vary within the mART group (589 [285–1558], 433 [10–1190], and 550 [12–1459] copies/10^6^ PBMC at T0, T48, and T96, respectively; p = 0.725), while they decreased significantly in the cART-treated patients (398 [156–1470], 200 [140–565], and 24 [10–270] copies/10^6^ PBMC at T0, T48, and T96, respectively; p = 0.006) (Fig 4). At the end of follow-up, levels of EBV-DNA was higher in the mART than in cART group (Fig 4).

## Discussion

A body of evidence indicate that mART in HIV-1-infected patients is associated to a higher rate of intermittent viremia and viral rebound with respect to that observed in cART-treated patients [2, 4–7]. Although this would suggest that mART has a lower impact on HIV-1 reservoir, the data produced using HIV-1 DNA load on PBMC as a marker of HIV-1 reservoir size are controversial [11–13].

In the present study, the HIV-1 reservoir size was estimated by measuring both HIV-1 DNA and intracellular HIV-1 RNA, a marker of ongoing viral activation/replication that may continuously refill the viral reservoir. During the study period, beyond individual patients’ variability, we found an overall slight decline of both markers in the cART group, whereas they remained fairly stable in the mART-patients. Thus, while cART treatment seemed to continuously reduce the size of the HIV-1 reservoir, confirming previous findings [25], a subliminal HIV-1 replication may contribute to refilling the HIV-1 reservoir in mART-treated patients. Chronic immune activation/inflammation is a hallmark of HIV-1 pathogenesis [10]. The findings that the percentage of both activated T and B cells rises in mART-treated patients, supports the hypothesis that mART has a lower impact on the HIV-1 reservoir. Since memory T cells are the preferential target for HIV-1 their activation may contribute to maintaining the viral reservoir by both activating HIV-1 replication and expanding latently infected cells [26]. A potential limitation of this study was the sole use of the CD38 marker on CD8 T cells to estimate their activation, and, unfortunately, the limited number of cells did not allow us to analyse the status of CD4 T cell activation/proliferation, even with other markers.

The activation of T and B cells is mainly triggered by microbial products. Indeed, pathogen-associated molecular patterns (PAMPs) and damage-associated molecular patterns (DAMPs) induce a potent innate immune response through the engagement of several Toll-like receptors (TLRs), which lead to T and B activation [27]. Notably, we did not find significant changes in PAMPs levels neither in the cART, as expected, nor in the mART-treated patients. Thus, the control of HIV-1 replication induced by mART may be sufficient to prevent the massive HIV-1-induced T-cell depletion that causes damage to the intestinal mucosa, promoting translocation of microbial products into circulation [27,28]. Interestingly, we found that in mART-treated patients there was an increase in levels of mtDNA. It has been suggested that NRTI-based cART is associated to diminished mitochondrial functions [29], as these drugs may interfere with mtDNA polymerase γ and interrupt mtDNA replication, leading to its depletion [30]. The presence of NRTIs may thus explain why cART-treated patients have lower mtDNA levels than patients treated only with PI. Once released from cells, mtDNA levels, significantly higher in mART-treated than in cART-treated patients, may substantially contribute to stimulating T and B cells.

To our knowledge, this is the first time that activation of B cells has been investigated in mART-treated patients. Chronic B-cell stimulation and expansion of EBV-infected B-cells [31–34] may increase the risk of EBV-related malignancies [16, 35, 36]. The hyperactivation of B cells by HIV-1 is characterized by a higher expression of activation markers (CD80 and CD86) [31] and of activated memory B cells (CD21^low^CD27^+^ B cells) which have undergone HIV-1-induced activation and differentiation to plasmablasts [24]. We found that levels of activated B-cell significantly increased over time in the mART-treated patients, while no differences were noted in the cART-treated ones. Moreover, while levels of EBV-DNA decreased in the cART group, they remained stable in the mART group. Thus, the control of immune activation status seems to play a role not only in reducing HIV-1 reservoir, but also in lowering EBV-DNA levels. By contrast, the mART, which is not as efficient as cART in controlling DAMPs levels and B-cell hyperactivation, may promote EBV reactivation and/or polyclonal expansion of EBV-infected B cells, favoring the onset of EBV-related malignancies.

In conclusion, the results of the study outlined here show that mART has a lower control on HIV-1 reservoir and immune activation than cART. The higher risk of intermittent viremia or virological failure episodes in mART strategy may be due to a persistent perturbation of the immune system in response to a persistent replication of HIV-1 reservoir and *vice versa*. These findings underline the fact that patients treated with mART should be studied not only for classical parameters, i.e. HIV-1 plasmaviremia and CD4 cell count, but also for HIV-1 reservoirs and immune activation. Moreover, the long-term monitoring of B-cell activation is important to avoid the risk of EBV-related malignancies. We are aware that limitation of this study is the low number of patients studied; a larger clinical studies are warranted to confirm these results.
